# Letters of Advice

**Published:** 1859-11

**Authors:** 


LETTERS OF ADVICE.—It has been our lot, during the last twenty years, to receive an amount of letters of this kind—enough to fill the mouth of the crater of Vesuvius, and all the money we have obtained for our answers would not, all told, we are sure, enable us to buy a first-rate horse and buggy. If the time thus spent could be correctly estimated it could not possibly be found to be less than six or eight months, involving a vast amount of toil and inconvenience, to say nothing of expense, which, in the aggregate, must have been very great. We recollect paying for one letter, when the postage was twenty-five cents, half a dollar. It covered nearly ten pages of foolscap, giving the most minute and circumstantial details of a case that was not of the slightest interest to us in any way. Of course, that letter was sent to Gehenna long before one-half of it was read. To have done otherwise would have been impossible. If only our *friends* knew how these letters annoy and worry us, they would not, we are sure, so constantly inflict them upon us. What are their patients to us, that we should be compelled to read the tedious accounts of their cases, and, worse than all, answer their selfish lucubrations? Do they suppose that we have no other business? Verily, verily, gentlemen, we say unto you that such messengers are tolerable only when they inclose a proper fee. In England, and indeed throughout all decent Christendom, that fee is never less than five dollars, from which it ranges, in many cases, as high as twenty-five. The sight of a bank-note is always agreeable to one who is compelled to earn his living by the sweat of his brow; but especially is it cheering to him whose eyes and hands are weary and aching with the labor of writing books and furnishing materials for the pages of a medical journal. Besides, how can a man possibly discern the strong points in such a letter if he is not properly remunerated for his services? The expectation of a fee always wonderfully quickens a physician's sympathy and clearness of perception. We never answer these charity-letters without a feeling of distrust that our advice may be anything but salutary. If we had a benevolence as diffusive as that of a Howard we should rebel at labor and annoyance so ungenerously and so inconsiderately imposed upon us by the profession. A physician has no more right to ask such services gratuitously for his patients than he has to send for his professional brother in a case of ordinary consultation without the prospect of reward, when the patient is amply able to pay. A reasonable fee should always accompany the letter. The only exception to this rule is where a practitioner solicits advice for himself or some member of his family, or for a very destitute person.

